# Immobilization of *Paenibacillus polymyxa* with biopolymers to enhance the production of 2,3-butanediol

**DOI:** 10.1186/s12934-024-02633-5

**Published:** 2025-01-10

**Authors:** Jnanada Joshi, Sarah Vanessa Langwald, Olaf Kruse, Anant Patel

**Affiliations:** 1 Hochschule Bielefeld – University of Applied Sciences and Arts (HSBI), Bielefeld, Germany; 2 Bielefeld University, Bielefeld, Germany

**Keywords:** *Paenibacillus polymyxa*, 2,3-butanediol, Immobilization, Alginate, Carrageenan, Chitosan

## Abstract

**Background:**

*Paenibacillus polymyxa*, is a Gram-positive, plant growth promoting bacterium, known for producing 98% optically pure 2,3-butanediol, an industrially valuable chemical for solvents, plasticizers and resins. Immobilization of *Paenibacillus polymyxa* has been proposed to improve the cell stability and efficiency of the fermentation process, reduce contamination and provide easy separation of butanediol in the culture broth as compared to conventional bioprocesses. This research aimed to explore the potential of *Paenibacillus polymyxa* with immobilization technique to produce 2,3-butanediol.

**Results:**

We investigated different immobilization methods with natural biopolymers like alginate, chitosan and carrageenan-chitosan-based immobilization. These methods were further investigated for their immobilization efficiency and yield in 2,3-butanediol production. Carrageenan-chitosan beads enabled a higher cell concentration and demonstrated superior cell retention to calcium-alginate-chitosan beads. Carrageenan-chitosan immobilization preserved 2,3-butanediol production in bacteria and increased the product formation rate.

**Conclusion:**

Carrageenan-chitosan immobilization enables non-pathogenic *Paenibacillus polymyxa* to be a capable 2,3-butanediol producer with increased product formation rate, which has not been previously reported. This novel strategy offers promising alternative to traditional fermentation processes using pathogenic strains and can be further applied in co-cultivations for metabolite production, wastewater management and bioremediation.

**Supplementary Information:**

The online version contains supplementary material available at 10.1186/s12934-024-02633-5.

## Background

The biotechnological production of 2,3-butanediol (2,3-BDL) through bacterial fermentation is potentially valuable with varied industrial applications as fuel [1], bulk chemical for polymers [2, 3], in cosmetics and personal care industries [4], agriculture [5–7] and pharmaceuticals [8]. Known bacterial BDL producers include *Enterobacter aerogenes*,* Klebsiella pneumonia*,* Klebsiella oxytoca*,* Serratia marcescens*,* Bacillus licheniformis* and *Paenibacillus polymyxa* [9]. So far, the highest 2,3-BDL yields 117.4 g/L [10] and 148 g/L [11] have been reported by *Enterobacter*,* Klebsiella* and *Serratia* [12]. Interest in the bacterium *P. polymyxa* as a large-scale 2,3-BDL producer is rapidly increasing due to its non-pathogenicity and ability to utilize wider range of carbon sources which is advantageous for lignocellulosic biomass as feedstock [13]. Known plant growth-promoting bacterium, *P. polymyxa* can produce significant amounts of 2,3-BDL, which can be utilised for production of solvents, plasticizers and resins [14]. Plant growth-promoting bacterium *Paenibacillus brasilensis* produces 2,3-BDL, with some strains capable of producing up to 27 g/L in 72 h [15]. However, microbial production of high 2,3-BDL concentrations and high product yields require expensive substrates such as glucose, leading to uneconomical industrial scale production [16]. *P. polymyxa* is further known to form exopolysaccharides (EPS) which are detrimental to 2,3-BDL fermentation pathway, as they reduce saccharides conversion to 2,3-BDL and increase the viscosity in the medium [17]. Optimization of these cultivation processes and immobilization techniques can address these challenges to improve fermentation efficiency by alleviating substrate costs, EPS formation and low product yields.

Immobilization or microencapsulation involves encasing viable cells within a gel matrix with permeable membranes [18, 19]. It enables easy handling and application, protection from biotic and abiotic factors, longer shelf life, controlled release and increased efficacy [20]. This method commonly employs various synthetic polymers like polyurethanes, polylactic acids, polycaprolactones and natural biopolymers like agarose, alginate, κ-carrageenan, chitosan and collagen. The biochemical parameters of alginate, agarose and κ-carrageenan are preferred to minimize cell damage [21]. Natural biopolymers formed in living organisms are popular for immobilization due to their good permeability for low-molecular substances and gases [22]. This microenvironment ensures the diffusion of nutrients and the removal of metabolic waste. Biopolymers have the additional benefit of being biodegradable and biocompatible [20, 23–25]. Immobilizing the microorganisms avoids direct contact and protects against shear forces in fermentation broth [20]. In recent years, immobilized cell systems have been commonly applied for biotechnological purposes, e.g., in bioremediation and biodegradation, biocontrol, pesticide application and the production of various metabolites [26]. For 2,3-BDL production, immobilization of *P. polymyxa* may improve fermentation stability and efficiency, reduce contamination risk and increase product concentration.

Previous optimization studies on enhancing 2,3-BDL production largely focused on medium components, fermentation conditions such as temperature, inoculum size, pH and aeration rates [27]. Co-cultivation strategies for enhancing 2,3-BDL production are also gaining interest, the co-cultivation of *P. polymyxa* and recombinant *E. coli* has been potentially applied to improve acetoin and 2,3-BDL production [28]. Immobilization of *P. polymyxa* in a protective matrix can improve the stability and efficiency of the fermentation process, reduce contamination and increase the concentration of butanediol in the medium [29]. Immobilization can also help to circumvent the problems associated with EPS formation. The most commonly employed immobilization methods include alginate-based immobilization, chitosan-based immobilization and polysaccharide-based immobilization. Cell leakage from the overgrowth of bacteria in the beads can be prevented by applying several coatings of biopolymers to make the beads more resistant to breakage [30].

The aim of the work presented here is to study the potential of immobilized *P. polymyxa* in producing 2,3-BDL with high productivity and high final product titre in fed-batch fermentation. We aim to develop a novel immobilization for bacteria with bio-based materials to influence growth and 2,3-BDL production. This immobilized bacterial bioprocess can be further applied in co-cultivations for metabolite production, wastewater management and bioremediation.

## Materials and methods

### Preculture and main culture

The preculture and main culture of *Paenibacillus polymyxa* ATCC 842 from DSMZ - German Collection of Microorganisms and Cell Cultures GmbH were cultivated in 100 mL flasks without baffles on a light shaker from Edmund Bühler with an illumination intensity of 670 µmol m^− 2^ s^− 1^ and shaking frequency of 120 rpm at 26 °C. The preculture served as an inoculum for the main culture. The total volume of all cultures reached 30 mL with PS medium (supplementary material) in 5 replicates.

### Chitosan-coated carrageenan beads

**Fig. 1:**
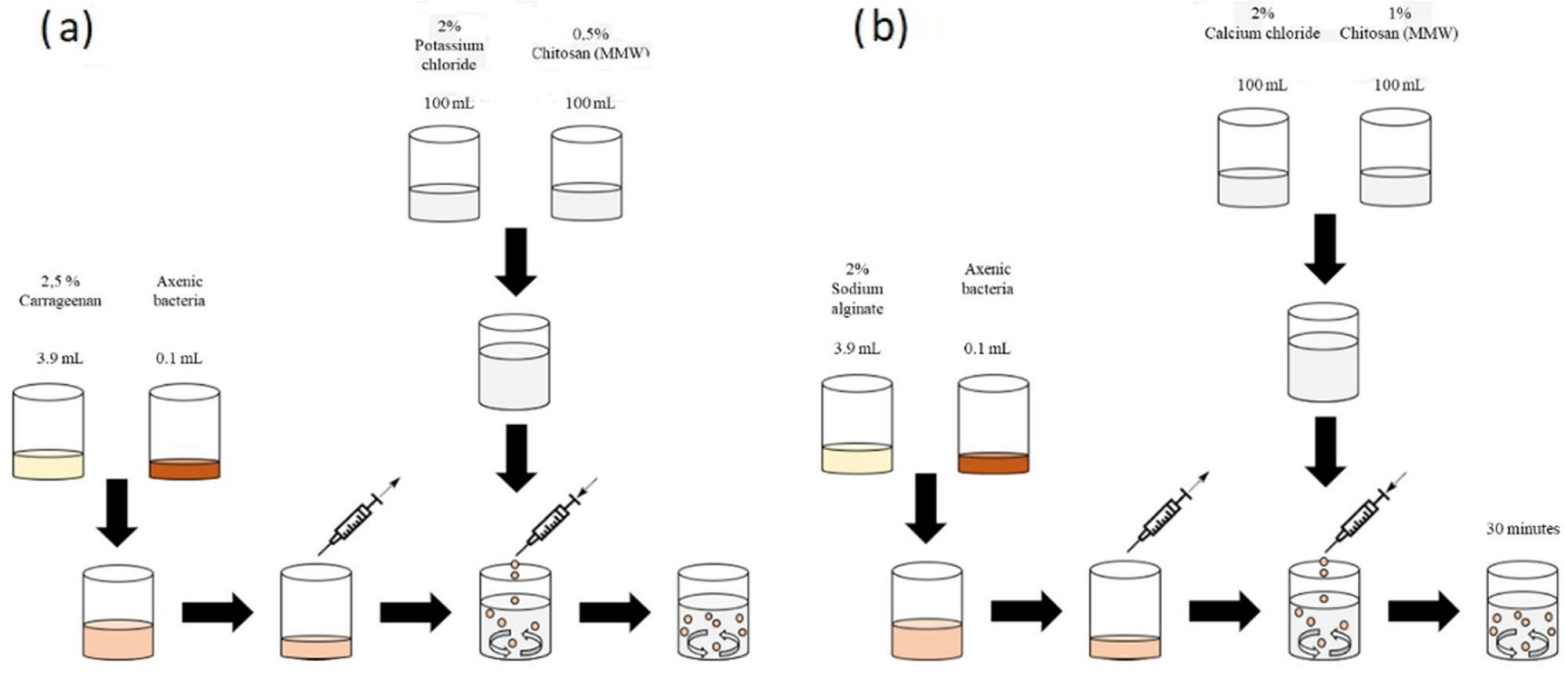
**a** Manufacturing process of chitosan-coated carrageen beads. **b** Manufacturing process of chitosan-coated calcium alginate beads

The complex coacervation method was applied to form the chitosan-coated carrageenan beads (Baruch [31]). 0.5% weight/volume (w/v) medium molecular weight chitosan was added to the cross-linking solution of 2% w/v potassium chloride (KCl). A drop-in process carried out the production of the chitosan-coated carrageenan beads (Fig. 1a). The collecting solution consisted of 100 mL 0.5% w/v chitosan and 100 mL 2% w/v KCl. These were mixed in a 25 mL beaker with a magnetic stirrer at 300 rpm. As a one-drop solution, 3.9 mL of 50 °C preheated 2.5% w/v κ-carrageenan and 0.1 mL of the bacterium were dropped into the collecting solution by a syringe (Braun Sterican, Germany 0.80*40 mm, 21G). The beads were stirred for two hours. Then they were poured through a strainer and washed with sterile ddH_2_O. By doing this, 40 beads with a diameter of 5 mm were produced.

### Chitosan-coated calcium alginate beads

**Fig. 2:**
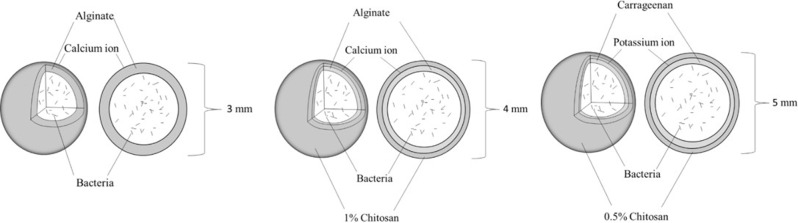
Production of uncoated vs. coated beads. Structure and photo of chitosan-coated calcium alginate beads and structure of uncoated (left) beads. Structure and photo of chitosan-coated carrageenan beads (below). The bead is coloured in grey to emphasize its layers; normally, beads are transparent

**Fig. 3:**
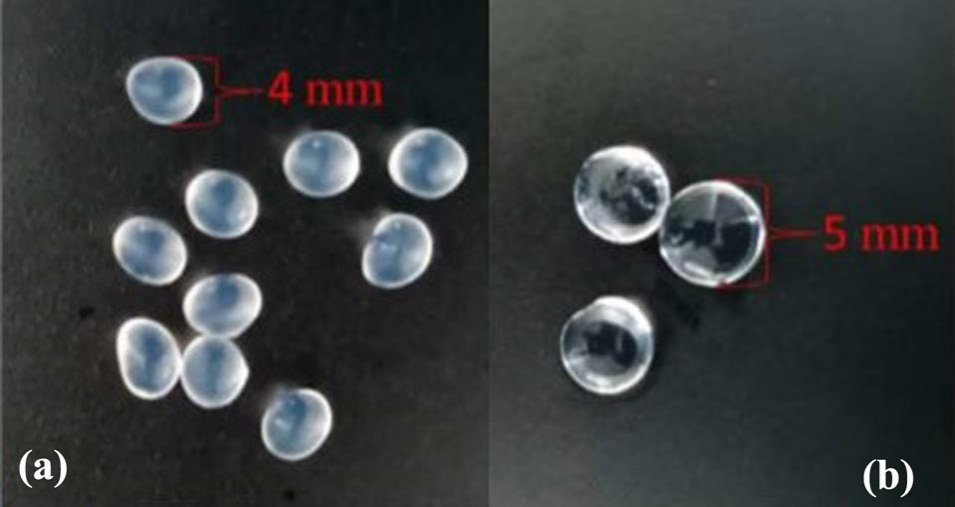
Uncoated **a** vs. coated beads **b**

Ionotropic gelation was applied to form the chitosan-coated calcium alginate beads based on optimizing previously known method (Baruch [31], Fig. 1b). In this process, 1% w/v chitosan was added to the cross-linking solution of 2% w/v calcium chloride (CaCl_2_). The polymer solution consisted of negatively charged 2% w/v sodium alginate. For the collecting solution, 100 mL 1% w/v chitosan was mixed with 100 mL 2% w/v CaCl_2_ in a 250 mL beaker with a magnetic stirrer and a stirrer rotation frequency of 300 rpm. A mixture of 3.9 mL sodium alginate and 0.1 mL bacteria was drawn up with a syringe (Braun Sterican, Germany 0.80*40 mm, 21G) and dropped into the solution. After 30 min, the produced beads were poured through a strainer and then washed with sterile ddH_2_O. By doing this, 40 beads with a diameter of 4 mm were produced (Figs. 2, 3).

### Calcium alginate beads

Ionotropic gelation was also applied to form the calcium alginate beads. The process is similar to the production of chitosan-coated calcium alginate beads, except that instead of 100 mL chitosan mixed with 100 mL CaCl_2_, only 200 mL CaCl_2_ was dropped into the collecting solution.

### Cell count

The cell count was determined with the help of the Bürker counting chamber. For determination of the cell concentrations in beads, the volumes of the beads were initially calculated according to the equation of the spherical volume: V_bead_ ≈ 4/3 π × r^3^.

The volume of the chitosan-coated calcium alginate beads, which had a radius of 2 mm, was 0.03351 mL. The chitosan-coated carrageenan beads had a volume of 0.06545 mL. The beads for the cell counts were treated with 1 mL of 1 M sodium citrate solution for dissolution, hence 1 mL was added to the calculated volumes, resulting in the total volume of each bead. To calculate the cell concentration, the following equation by Ableitner O. is applied [32].

$$\:{\text{c}}_{1}\times\:{\text{V}}_{1}={\text{c}}_{2}\times\:{\text{V}}_{2}$$

c_1_ = cell concentration in the bead [cells / mL]

c_2_ = cell concentration in the total volume [cells / mL]

V_1_ = volume of the bead [mL]

V_2_ = total volume [mL]

### Substrate analysis

For 2,3-BDL and glucose concentrations, 1 mL samples were removed during each cultivation and frozen at -28 °C. The determination was made by a HPLC (high performance liquid chromatography) system from VWR Hitachi Primaide 903 − 0799, Germany. The materials were separated by means of a reverse phase column Repromer H, 9 μm, 250 × 8 mm, Altmann Analytik, Germany [33]. The eluent applied to determine the glucose concentration was ddH_2_O with a volume flow was 0.7 mLmin ^− 1^ and oven temperature of the column set to 25 °C. The 2,3-BDL concentration in the samples was also determined with the same column. Here, a 6 mM sulfuric acid was applied as the eluent at room temperature. Both substrates were detected via the refractive index. The cultivation samples that had been frozen for analysis were thawed. The insoluble components were removed by centrifuging the samples at 14,000 rpm for 5 min. The samples were then diluted with ddH2O and transferred to HPLC vials. Quantification was carried out against high-purity reference standards of glucose and 2,3-BDL obtained from Carl Roth GmbH & Co. KG, Karlsruhe, Germany and Sigma-Aldrich, USA, respectively.

### Stability of the beads

The beads were studied for cell leakage and structural integrity with Atomic Force Microscopy (AFM), FlexAFM Axiom from Nanosurf GmbH, Germany. Coated and uncoated beads were checked for leakage on day 0, 5 and 7 respectively. The bead sample was precisely incised into 0.5–1 cm shavings. A suitable substrate like glass slide was selected, cleaned thoroughly and then the sample was carefully adhered to the substrate and finally the sample was properly dried before mounting on the AFM stage. The cantilever Tap190Al (Budget sensors, Bulgaria) was applied in tapping mode on FlexAFM Axiom from Nanosurf GmbH, Germany at room temperature, standard settings of setpoint 55%, P-gain 550, I-gain 1000, D-gain 100 and vibration amplitude 2 V with scan forward measuring 25 μm size.

### Oxygen measurement

The oxygen concentration in the flasks was determined by the Clark electrode (S1, Hansatech Instruments, UK) an electrochemical sensor for measuring the partial pressure of O_2_ gas in a sample. A voltage of 0.6 V was applied between the electrodes, a platinum cathode and a silver/silver chloride anode in a KCl solution and the current flow was measured [34].

First, the Clark electrode was calibrated by adding 2 mL ddH2O to the measuring cell, which was saturated with oxygen by shaking. The water now contained 258 µmol oxygen per liter. The zero value was determined by adding a spatula tip of sodium dithionite. The measuring cell was then rinsed twice with 2 mL ddH_2_O. The measurements were carried out for free and immobilized bacteria with a cell concentration of 1 × 10^4^ cells per mL. To determine the oxygen consumption rate of the free bacteria, 2 mL of cell suspension was pipetted into the measuring cell. To demonstrate the permeability of the beads to oxygen, 10 beads with immobilized bacteria were placed in the measuring cell with 2 mL of medium. This allowed the oxygen uptake rate of the beads to be determined.

### Growth of microorganisms

The specific growth rate µ was calculated to describe the increase in cell concentration in the growth phases. Furthermore, the specific substrate uptake speed $\:{\text{q}}_{\text{S}}$ and product formation rate $\:{\text{q}}_{\text{P}}$ analogous to the growth rate was described. The reaction rates for substrates were negative and positively defined for products. The following equation was applied:

$$\:{q}_{S}=\:\frac{1}{x}\frac{{d}_{S}}{{d}_{t}}$$

$$\:{q}_{P}=\:\frac{1}{x}\frac{{d}_{P}}{{d}_{t}}$$

*q*_*S*_ = specific substrate uptake rate [g g^−1^ d^−1^]

*q*_*P*_ = specific product formation rate [g g^−1^ d^−1^]

S = substrate concentration [g L^− 1^]

P = product concentration [g L^− 1^]

X = biomass concentration [g L^− 1^]

t = time [d]

For the mathematical representation of the product yield, $\:{Y}_{P/S}\:$in relation to the supplied substrate concentration can be related to the corresponding set speeds.

$$\:{Y}_{P/S}=\:\frac{{d}_{P}}{{d}_{S}}=\frac{{q}_{P}}{{q}_{S}}$$

*Y*_*P/S*_ = product yield [g g^−1^].

By applying the following equation by Takors R., we can calculate the biomass yield per substrate consumed [35].

$$\:{Y}_{X/S}=\:\frac{{d}_{X}}{{d}_{S}}=\frac{\mu\:}{{q}_{S}}$$

*Y*_*X/S*_ = biomass yield [g g^−1^]

## Experimental designs and statistical analyses

Each experiment was carried out in replicates of 5. For data of growth, the homoscedasticity of the data was tested and then analysed first by one-way ANOVA and then by least significant difference (LSD) post-hoc analysis, with significance set at *p* < 0.05. The statistical analyses were performed with IBM SPSS software, version 27.

## Results

### Comparison of bacteria immobilized in alginate and carrageenan beads coated with chitosan

**Fig. 4:**
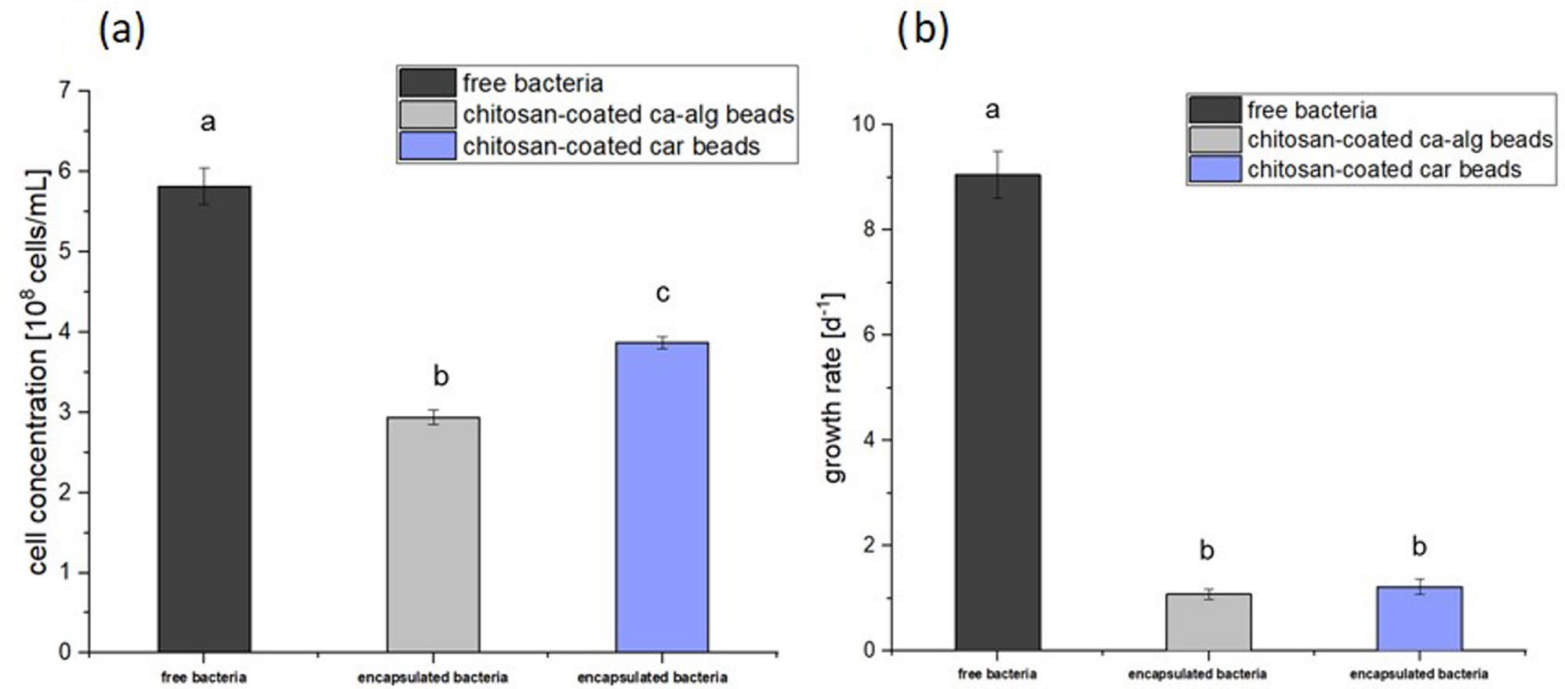
**a** Cell concentration of free and immobilized bacteria *n* = 5; mean $\:\pm\:\:$SD; one-way-ANOVA with Bonferroni’s post-hoc test, *p* < 0.05. **b** Growth rates of free and immobilized bacteria *n* = 5; mean $\:\pm\:\:$SD; one-way-ANOVA with Bonferroni’s post-hoc test, *p* < 0.05

**Table 1:** Substrate uptake rate of the immobilized bacteria vs. free bacteria

Cultivation in PS medium	Substrate uptake rate q_S_ (gS g_x_^−1^ d^− 1^)
Axenic free bacteria	2.48 ± 0.06
Immobilized bacteria in chitosan-coated alginate beads	0.92 ± 0.08
Immobilized bacteria in chitosan-coated carrageenan beads	0.97 ± 0.06

Figure 4a demonstrates that after 30 h, the cell concentrations of the immobilized bacteria were lower than the axenic free bacteria. Bacteria immobilized in calcium alginate reached a maximum cell concentration of 3.69 × 10^8^ ± 0.11 cells per mL with biomass of 3 ± 0.35 g^− 1^g_bead_^−1^d^− 1^ corresponding to biomass of 5 ± 0.18 g^− 1^g_bead_^−1^d^− 1^ for free bacteria. The maximum cell concentration of the bacteria immobilized in carrageenan was 4.75 × 10^8^ ± 0.13 cells per mL corresponding to biomass of 3.7 ± 0.41 g^− 1^g_bead_^−1^d^− 1^. Although carrageenan beads exhibited a 28.7% higher cell concentration compared to alginate beads, this difference was not statistically significant (one-way ANOVA: F(2,12) = 438.77, *p* = 0.587; Bonferroni’s post-hoc test, *p* < 0.05). This suggests that the observed variation falls within the expected biological range. Figure 4b illustrates that immobilization successfully restricted the growth rates as compared to the free bacteria. Specifically, chitosan-coated ca-alginate beads restricted the growth of immobilized cells by 738% and Chitosan-coated carrageenan beads by 641.8% relative to free bacteria.

### Comparison of cell retention of bacteria immobilized in alginate and carrageenan beads coated with chitosan

**Fig. 5:**
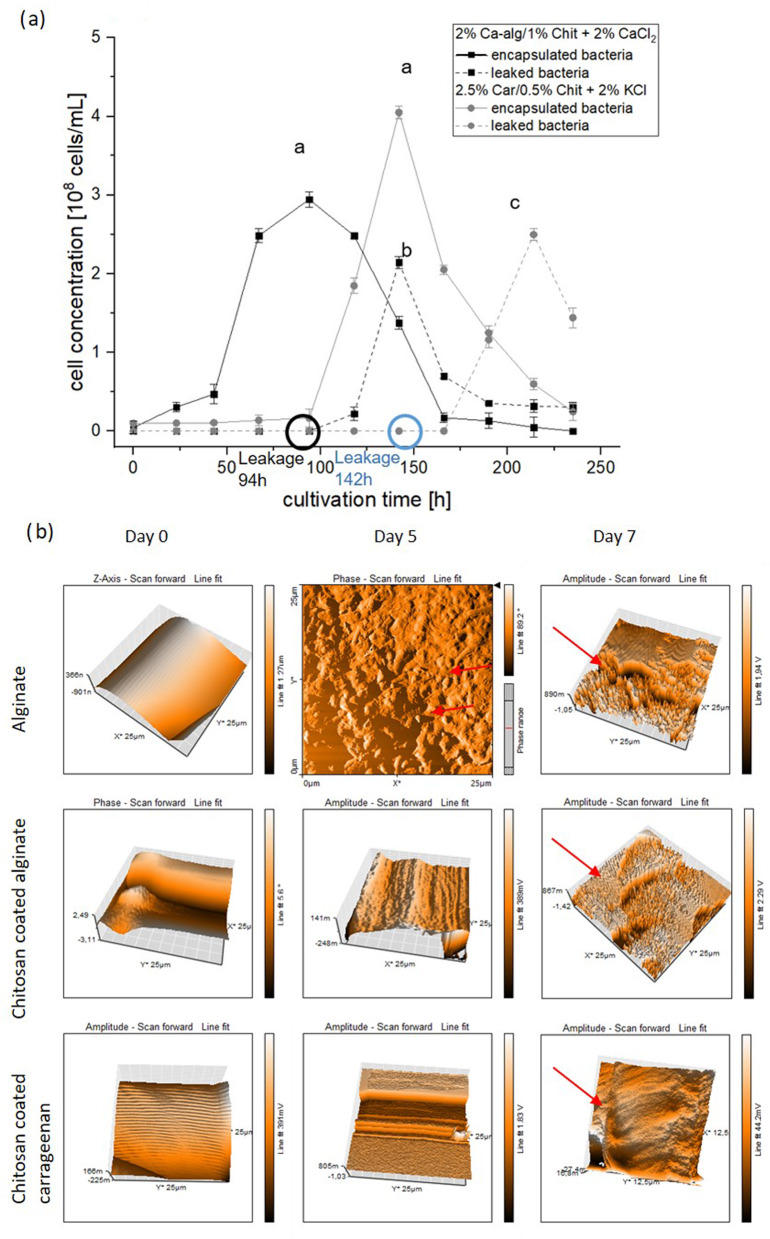
**a** Cell leakage from beads *n* = 5; mean $\:\pm\:\:$SD; RM-ANOVA with Bonferroni’s post-hoc test, *p* < 0.001. the marked circles indicate the cell leakages from bead systems corresponding to increase of leaked bacteria in the medium. **b** AFM studies on the structural integrity of chitosan-coated alginate and carrageenan beads at days 0, 5 and 7. Day 0- smooth, intact bead surfaces loaded with bacteria, Day 5- red arrows showing cells on surface of alginate beads already, no cells seen on coated beads, Day 7- Alginate and chitosan coated beads showing disintegration, carrageenan coated bead still intact. Uncoated alginate beads: Cells visible on the bead surface by day 5 (**b**, highlighted with arrows), Complete bead rupture observed by day 7. Coated beads: Demonstrated superior cell retention compared to uncoated beads

The stability of chitosan-coated calcium-alginate and carrageenan beads was investigated with varying molecular weights and concentrations of chitosan (supplementary material attached). We aimed to identify the corresponding biopolymer concentrations for the most stable and robust bead formulations for BDL production. Beads were incubated on a rotary shaker at 120 rotations/minute, 26°C over time to assess their stability. The results, as illustrated in Fig. 5a and b, reveal distinct differences in bead performance. The results in Fig. 5a demonstrate that in the case of beads made with low molecular weight chitosan, early leakage could be observed. The beads with medium molecular chitosan, however, showed no leakage and were stable for up to 98 (Ca-alg / Chit) or 107 days (Car / Chit), respectively. These results indicate that indeed the molecular weight of chitosan significantly influences bead stability, consistent with previous research [36].

Atomic Force Microscopy (AFM) studies of the beads provided further evidence supporting these experimental results. AFM studies corroborated the previous result that coated beads prevent cell leakage better than the uncoated beads by retaining cells. Cells could be seen on the surface of the alginate bead by day 5 (Fig. 5b highlighted with arrows) and beads were ruptured by day 7. Carrageenan coated beads showed the best structural integrity as compared to the other two bead systems. Notably, carrageenan-chitosan beads demonstrated superior longevity and structural integrity compared to other bead formulations which could be particularly beneficial for extended-duration applications and improved cell retention. These results highlight the importance of the molecular weight of the biopolymer and their concentration in bead compositions to optimize bead stability, cell retention and overall structural integrity in immobilization applications. AFM results further add weight to these results by providing valuable visual confirmation.

### Production of 2,3-butanediol from bacteria immobilized in alginate and carrageenan beads coated with chitosan

**Fig. 6:**
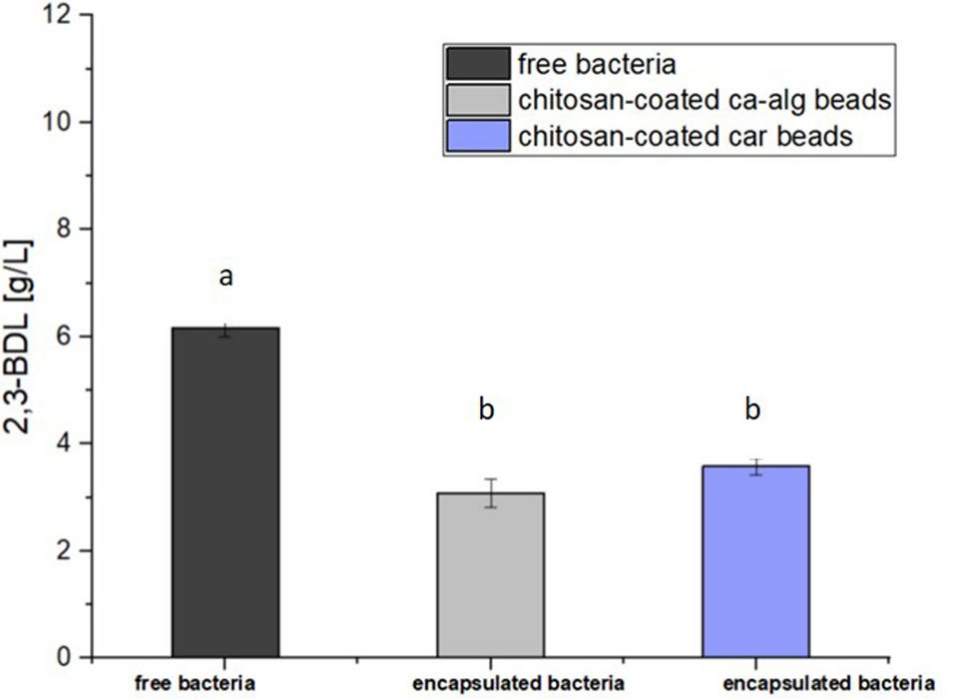
Butanediol production one-way ANOVA with Bonferroni’s post-hoc test *p* < 0,05 for qP F2,19 = 1,87; *p* = 0,185

We investigated the influence of bacterial immobilization on 2,3-butanediol (2,3-BDL) production, comparing immobilized bacteria with free bacteria. The results, as depicted in Fig. 6, reveal an interesting insight. The maximum 2,3-BDL concentration of calcium alginate immobilized bacteria were 3.08 ± 0.21 g/L as seen in Fig. 5a. The bacteria immobilized with carrageenan provided maximum 2,3-BDL concentration of 3.58 ± 0.14 g/L. With the 2,3-BDL concentrations of both types of beads compared with each other, it was observed that the carrageenan beads deliver a 16.2% higher 2,3-BDL production. However, no significant difference was seen in the statistical analysis (one-way ANOVA F 1.19 = 146.12; *p* = 0.325 with Bonferroni’s post-hoc test at *p* < 0.05). Comparing the 2,3-BDL concentrations of the immobilized bacteria, more product was detected to those of free bacteria. According to one-way ANOVA, these results are statistically significant (F 1.19 = 146.12; *p* < 0.001 with Bonferroni’s post-hoc test at *p* < 0.05). The product formation rate q_P_ (gP g_x_^−1^d^− 1^), product yield Y_p/S_ (g_P_g^s−1^) and product per 10^8^cells were further calculated for a more detailed insight into the influence of the immobilization on the 2,3-BDL production. These calculations provide deeper understanding of the production efficiency in immobilized systems compared to free bacteria. The calculated product formation rate and product yield of the immobilized bacteria were lower than the axenic free bacteria after 30 h. Bacteria immobilized in chitosan-calcium alginate showed a product formation rate of 0.25 ± 0.007g_P_gx^− 1^d^− 1^ with product yield of 0.18 ± 0.05g_P_gS^− 1^ corresponding to product formation rate of 0.41 ± 0.14g_P_gx^− 1^d^− 1^ with product yield of 0.31 ± 0.03g_P_gS^− 1^ for free bacteria.

The product formation rate of the bacteria immobilized in carrageenan was 0.26 ± 0.06g_P_gx^− 1^d^− 1^ with product yield of 0.20 ± 0.04g_P_gS^− 1^. The product formation rate and yield of immobilized bacteria were lower than free bacteria, however, further investigation revealed that the butanediol production per cell of chitosan coated beads at 1.04 g/L/cell was indeed comparable to that 1.05 g/L/cell of free cells. This particularly means that the immobilization bioprocess was indeed efficient and that immobilization preserves 2,3-BDL production in bacteria with potential for enhancement. Overall calculated production rates may be lower in immobilized systems however, the per-cell efficiency remains comparable to free bacteria. These results highlight the potential of immobilization methods, particularly with carrageenan, for improving production efficiency in industrial 2,3-BDL production. Further research into optimizing these immobilization techniques could lead to significant advancements in industrial 2,3-BDL production.

### Comparison of oxygen consumption from bacteria immobilized in alginate and carrageenan beads coated with chitosan

**Fig. 7:**
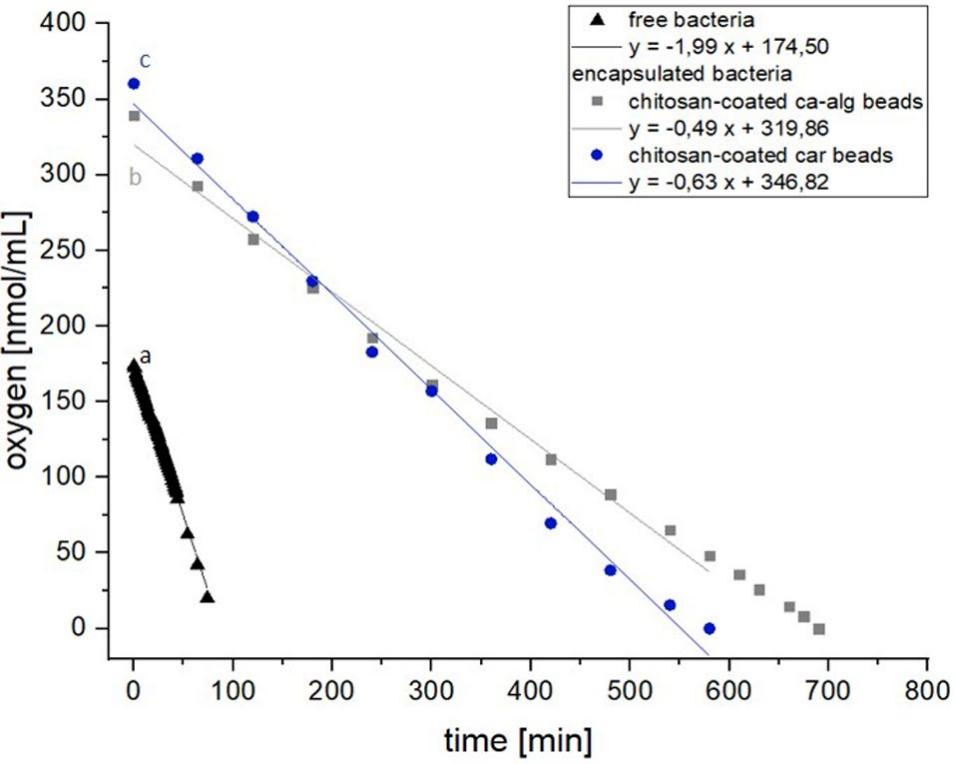
Oxygen uptake rates of immobilized bacteria vs. free bacteria one-way ANOVA F2.14 = 4198.91; *p* < 0.001 with Bonferroni’s post-hoc test at *p* < 0.05)

The substrate and oxygen diffusion limitation are often dependent on high cell concentration in the bead and bead size [36]. Bead size is important because it causes diffusional limitations of nutrients into cells and product exit from cells [37]. Figure 7 illustrates the oxygen concentrations of the cultures of free and immobilized bacteria over time. A regression line drawn through the individual measuring points with the slope correspond to the oxygen uptake rate. To determine the oxygen uptake rate per cell, the calculated gradient was divided by the cell concentration, as shown in Fig. 4a. The calculated oxygen uptake rates per cell listed in Table 2 show that the oxygen uptake rate of the immobilized bacteria was 3.9-fold (Ca-alg / Chit beads) or 3- fold (car / chit beads) lower than those of the free bacteria.

When comparing the two types of beads, carrageenan-chitosan beads demonstrated a statistically significant 32.8% higher oxygen uptake rate than calcium alginate-chitosan beads (one-way ANOVA F 2.14 = 4198.91; *p* < 0.001 with Bonferroni’s post-hoc test at *p* < 0.05). These results suggest that both types of beads reduce oxygen uptake compared to free bacteria however, carrageenan-chitosan beads provide superior oxygen transfer characteristics. This improved oxygen consumption could potentially lead to enhanced metabolic activity and product formation in immobilized cells. The observed differences in oxygen uptake rates between free and immobilized bacteria, as well as between different bead types, highlight the importance of optimizing immobilization materials and bead properties for specific bioprocess applications. Further research into the connection between bead composition, size and oxygen transfer could lead to significant improvements in the efficiency of immobilized cell systems for varied biotechnological processes.

**Table 2:** Oxygen uptake rates of immobilized bacteria vs. free bacteria

Cultivation	Oxygen uptake rate [nmol/cell*min]
Free bacteria	4.67 × 10^− 5^ ± 0.08 a
Immobilized bacteria in Chitosan-coated calcium alginate beads	1.19 × 10^− 5^ ± 0.05 b
Immobilized bacteria in Chitosan-coated carrageenan beads	1.58 × 10^− 5^ ± 0.07 c

*n* = 5; mean ± SD; one-way ANOVA with Bonferroni’s post-hoc test, *P* < 0.05

## Discussion

Current strategies for microbial fermentation of butanediol production have achieved significant success, with wild-type *Klebsiella pneumoniae* producing up to 150 g/L of 2,3-BDL at a productivity of 3.95 g L⁻¹ h⁻¹ [10]. Advances into the biosynthetic pathway understanding have enabled metabolic engineering strategies for enhancing 2,3-BDL production by overexpressing key enzymes [38]. Overexpression of budA and budB genes in *K. Pneumoniae* increased 2,3-BDL titer by 1.6-fold to 101.53 g/L after just 40 h of fermentation [39]. Comparatively, immobilization of bacteria offers advantages such as enhanced stability, simplified downstream processing and suitability for continuous operations [40]. However, immobilized systems face challenges including mass transfer limitations, reduced metabolic activity and scaling up difficulties for industrial production [40]. Amongst other bacterial candidates, *Paenibacillus polymyxa* is rapidly emerging as a promising 2,3-BDL producer due to its ability to utilize various carbon sources and its robustness in fermentation. Despite its promise in green energy applications, industrial-scale production remains economically challenging due to high glucose consumption [14, 41]. While current free-cell fermentation strategies generally offer higher production titers and rates, immobilization is advantageous for long-term continuous production or when simplified downstream processing is crucial [40]. Research on immobilizing *P. polymyxa* has demonstrated improvements in fermentation efficiency and product yields; immobilization of *P. polymyxa* enhanced 2,3-BDL production by shielding the bacterium from environmental shear stress and facilitating controlled metabolite release, improved acid tolerance observed in immobilized cells with glycerol as carbon source [42]. Similarly, improved resistance to osmotic stress and substrate inhibition in immobilized cells was also observed [28]. Immobilized cells have shown higher tolerance to substrate inhibition and prolonged fermentation lifespan leading to improved 2,3-BDL productivity compared to free cells [43]. Overall, these studies highlight the potential of immobilization as a promising strategy for bacterial 2,3-BDL production. However, the efficiency of immobilization may vary on biopolymers, media constituents, fermentation conditions and chosen immobilization method.

Our research paper supports this growing body of immobilization research by introducing a novel approach with coated beads of carrageenan-chitosan for *P. polymyxa* immobilization. We observed that 2,3-BDL production per cell was preserved in immobilized bacteria comparable to free cells. This result signified the potential of immobilization techniques to maintain productivity with additional benefits such as easier separation and downstream processing. Our results align with previous studies showing the effectiveness of both carrageenan and alginate as immobilization matrices. Enhanced mechanical stability and improved 2,3-BDL production with alginate and carrageenan immobilization has already been observed [44, 45]. Our research extends these results by exploiting a novel coating technique, combining carrageenan with chitosan to form beads with improved properties. Atomic Force Microscopy (AFM) results reveal that medium molecular chitosan-coated calcium-alginate and carrageenan beads exhibited stronger structures and fewer pores, even after an extended period. Chitosan coated carrageenan beads showed a more open and porous structure compared to alginate beads, potentially enabling a higher cell load and longer cell retention. Carrageenan can be enzymatically degraded by certain bacteria into oligosaccharides, potentially serving as a carbon source [46]. We hypothesise that this could support bacterial growth and metabolism, indirectly promoting 2,3-BDL production. These oligosaccharides might act as signaling molecules or metabolic intermediates, potentially influencing bacterial metabolism and 2,3-BDL biosynthesis. Thus, chitosan coated carrageenan beads could be important for continuous fermentation bioprocesses and also be utilised in co-cultivation strategies with different microorganisms to further improve 2,3-BDL production. However, the immobilization efficiency may vary on the bioploymers, media and fermentation conditions. Our research demonstrates that both carrageenan and alginate immobilization could positively influence the biosynthesis of 2,3-BDL by *P. polymyxa* under mentioned fermentation conditions. Costs, availability and specific application requirements could also influence the choice between carrageenan and alginate biopolymers for immobilization. Here, our immobilization of *P. polymyxa* utilising novel coated beads, presents a viable option for enhancing 2,3-BDL production at an industrial scale. The preservation of 2,3-BDL production per cell in immobilized bacteria and improved structural properties of carrageenan-chitosan beads provides promising avenues for optimizing bioprocesses. Future research will elucidate the underlying mechanisms of immobilization for industrial scale up. Development of efficient immobilization techniques for *P. polymyxa* can play a key role in advancement of bacterial production of 2,3-BDL in green energy solutions.

## Conclusion

This research highlights the significant potential of immobilized *Paenibacillus polymyxa* as a highly advantageous, non-genetically modified and cost-effective approach for optimizing 2,3-butanediol production. Our research presents immobilization of *Paenibacillus polymyxa* with novel chitosan coated carrageenan beads as a promising alternative to traditional production strategies, laying a strong foundation for future studies to elucidate the biopolymeric immobilization of *P. polymyxa* for sustainable 2,3-BDL production processes.

## Electronic supplementary material

Below is the link to the electronic supplementary material.

Supplementary Material 1

## Data Availability

No datasets were generated or analysed during the current study.

## Declarations

### Author information

JJ is currently a doctoral candidate at Bielefeld University and part of the working group “Fermentation and Formulation of Biologicals and Chemicals” at the University of Applied Sciences Bielefeld (HSBI). As part of her doctoral research, JJ developed scalable, carbon-neutral fermentation processes with innovative co-cultivation bioprocesses for biofuel applications. SVL was also affiliated as a Postdoc with the working group “Fermentation and Formulation of Biologicals and Chemicals” at the University of Applied Sciences Bielefeld (HSBI). OK is a Full Professor at the Department of Biology of Bielefeld University and the Scientific Director of the Center for Biotechnology (CeBiTec). OK leads the Algae Biotechnology Group, focusing on the molecular biology and synthetic biology of microalgae for biotechnological applications. AP is a Full Professor and Vice President for Research and Development at the University of Applied Sciences Bielefeld (HSBI). AP leads the Working Group “Fermentation and Formulation of Biologicals and Chemicals” focusing on various aspects of biotechnology including immobilization projects, fermentation processes and the development of bioproducts for agricultural applications.
