# Supplementary material for: Immobilization of Paenibacillus polymyxa with biopolymers to enhance the production of 2,3-butanediol
Source: Microb Cell Fact. 2025 Jan 10;24:15. doi: 10.1186/s12934-024-02633-5 (PMC11724508; doi:10.1186/s12934-024-02633-5)
Supplement: Supplementary file 1 — Supplementary Material 1 [file 12934_2024_2633_MOESM1_ESM.docx]

**SUPPLEMENTARY DATA**

## **Materials and methods**

## PS Medium: Composition of the PS medium with the respective concentrations and sources of supply.

| **Component** | **Concentration [L^-1^]** | **Source of supply** |
| --- | --- | --- |
| Glucose | 20g | Carl Roth GmbH & Co. KG, Karlsruhe, Germany |
| Yeast extract | 5g |  |
| Tryptone | 5g |  |
| ${MgSO}_{4}\times H_{2}O$ | 0,2g |  |
| ${{(NH}_{4})}_{2}SO_{4}$ | 3g |  |
| ${KH.}_{2}{PO.}_{4}$ | 3,5g | AppliChem GmbH,  Darmstadt, Germany |
| $K_{2}H{PO.}_{4}$ | 2,75g |  |
| Trace elements | 90µL | see table below |

Composition of the trace elements with the respective concentrations and supply source.

| **Component** | **Concentration [L^-1^]** | **Source of supply** |
| --- | --- | --- |
| ${FeSO}_{4}$ | 40mg | Carl Roth GmbH & Co. KG, Karlsruhe, Germany |
| $H_{3}{BO.}_{3}$ | 80mg | AppliChem GmbH,  Darmstadt, Germany |
| ${CuSO}_{4}\times5H_{2}O$ | 4mg | Carl Roth GmbH & Co. KG, Karlsruhe, Germany |
| ${{Na}_{2}MoO}_{4}\times2 H_{2}O$ | 4mg | Merck KgaA, Darmstadt, Germany |
| ${MnCl}_{2}\times4 H_{2}O$ | 500mg |  |
| ${ZnSO}_{4}\times7 H_{2}O$ | 10mg | Carl Roth GmbH & Co. KG, Karlsruhe, Germany |
| ${{Co(NO}_{3})}_{2}\times6 H_{2}O$ | 8mg | Merck KgaA, Darmstadt, Germany |
| ${CaCl}_{2}\times2 H_{2}O$ | 100mg | Carl Roth GmbH & Co. KG, Karlsruhe, Germany |
| Biotin | 1mg |  |

*Results*

*Comparison of cell retention of bacteria immobilized in alginate and carrageenan beads coated with chitosan*

Different molecular weights and concentrations of biopolymers were tested for bead production as described in materials and methods. Beads were selected based on produced shape, structure, thickness.

Diameter of the chitosan-coated Ca-alg microcapsules at the beginning and at the time of leakage, left 2% Ca-alg/1% Chit (MMW) + 2% CaCl2, right 2% Ca-alg/3% Chit (LMW) + 2% CaCl2.

Diameter of the chitosan-coated Car microcapsules at the beginning and at the time of leakage, left 2.5% Car/0.5% Chit (MMW) + 2% KCl, right 2.5% Car/3% Chit (LMW) + 2% KCl.
